# Severe Rheumatic Mitral Stenosis Presenting as Recurrent Hemoptysis in a Young Female With Respiratory Symptoms

**DOI:** 10.7759/cureus.110054

**Published:** 2026-06-01

**Authors:** Ahmed Osman, Aisha Alzahmi, Baher Zidan

**Affiliations:** 1 Internal Medicine, Sheikh Khalifa Medical City (SKMC), Abu Dhabi, ARE; 2 Emergency Medicine, Sheikh Khalifa Medical City (SKMC), Abu Dhabi, ARE

**Keywords:** hemoptysis, mitral stenosis, pulmonary hemorrhage, rheumatic heart disease, valvular heart disease

## Abstract

Rheumatic mitral stenosis remains a significant cause of cardiovascular morbidity in young adults worldwide, yet its diagnosis can be challenging when clinical presentation overlaps with concurrent infections. We present a case of a woman in her 30s who presented with dyspnoea, fever, and haemoptysis, initially attributed to community-acquired pneumonia with concurrent viral and bacterial infection. She received appropriate treatment and was discharged; however, her symptoms persisted, and she developed alveolar haemorrhage, prompting further investigations, which ultimately revealed severe mitral stenosis secondary to rheumatic heart disease. This case underscores the diagnostic complexity of mitral stenosis in the setting of overlapping pulmonary infections and highlights the critical importance of maintaining a broad differential diagnosis and pursuing thorough cardiac assessment in patients with unexplained haemoptysis.

## Introduction

Rheumatic heart disease (RHD) remains a major global health burden despite overall declines in incidence in high-income countries. It continues to cause substantial morbidity and mortality worldwide, particularly in endemic regions. In 2023, an estimated 14.5 million disability-adjusted life years (DALYs) were attributable to RHD globally [1].

Mitral stenosis (MS) is the most common chronic and debilitating valvular complication of RHD. The condition predominantly affects women, accounting for approximately 80% of cases. Clinical presentation varies geographically; patients in endemic regions typically present at younger ages, ranging from adolescence to the third decade of life, with pliable non-calcified valves characterized by commissural fusion. In contrast, patients in low-prevalence regions generally present later in life, between 50 and 70 years of age, with heavily calcified and fibrotic valves [2]. In MS, progressive narrowing of the mitral valve orifice leads to elevated left atrial pressure and subsequent pulmonary venous hypertension. This can result in pulmonary edema, alveolar hemorrhage, and hemoptysis, which are symptoms that may closely mimic primary respiratory infections, particularly when concurrent infection is present. Although hemoptysis is a recognized manifestation of MS secondary to elevated pulmonary venous pressure, it is an uncommon presenting symptom [3], and such atypical presentations in modern practice can lead to significant diagnostic delay, especially in regions with diverse patient populations where RHD may not be immediately considered.

## Case presentation

A woman in her 30s from the Philippines, previously not known to have any medical illnesses, presented to the emergency department with a three-day history of fever, worsening dyspnea, and hemoptysis. Six months prior, she had noticed progressive exertional dyspnea and unintentional weight loss of 8 kg, but had not sought medical attention. On further questioning, she reported reduced exercise tolerance, with dyspnea occurring while performing routine household chores, such as cleaning, corresponding with New York Heart Association (NYHA Classes II-III). Three days before presentation, she developed fever, night sweats, and up to four episodes of hemoptysis daily, the first involving frank blood with clots, with subsequent episodes showing blood-streaked yellow sputum.

On initial assessment, the patient was febrile with a temperature of 38°C, blood pressure of 110/64 mmHg, heart rate of 105 beats/minute with a regular rhythm, respiratory rate of 22 breaths/minute, and oxygen saturation of 98% on room air. She appeared mildly distressed on examination. Cardiovascular examination revealed tachycardia with a regular rhythm and no audible murmurs. Jugular venous pressure was not assessed. Respiratory examination demonstrated bilateral crackles over the lower lung zones. No peripheral edema was noted; the remainder of the examination was unremarkable.

**Figure 1:**
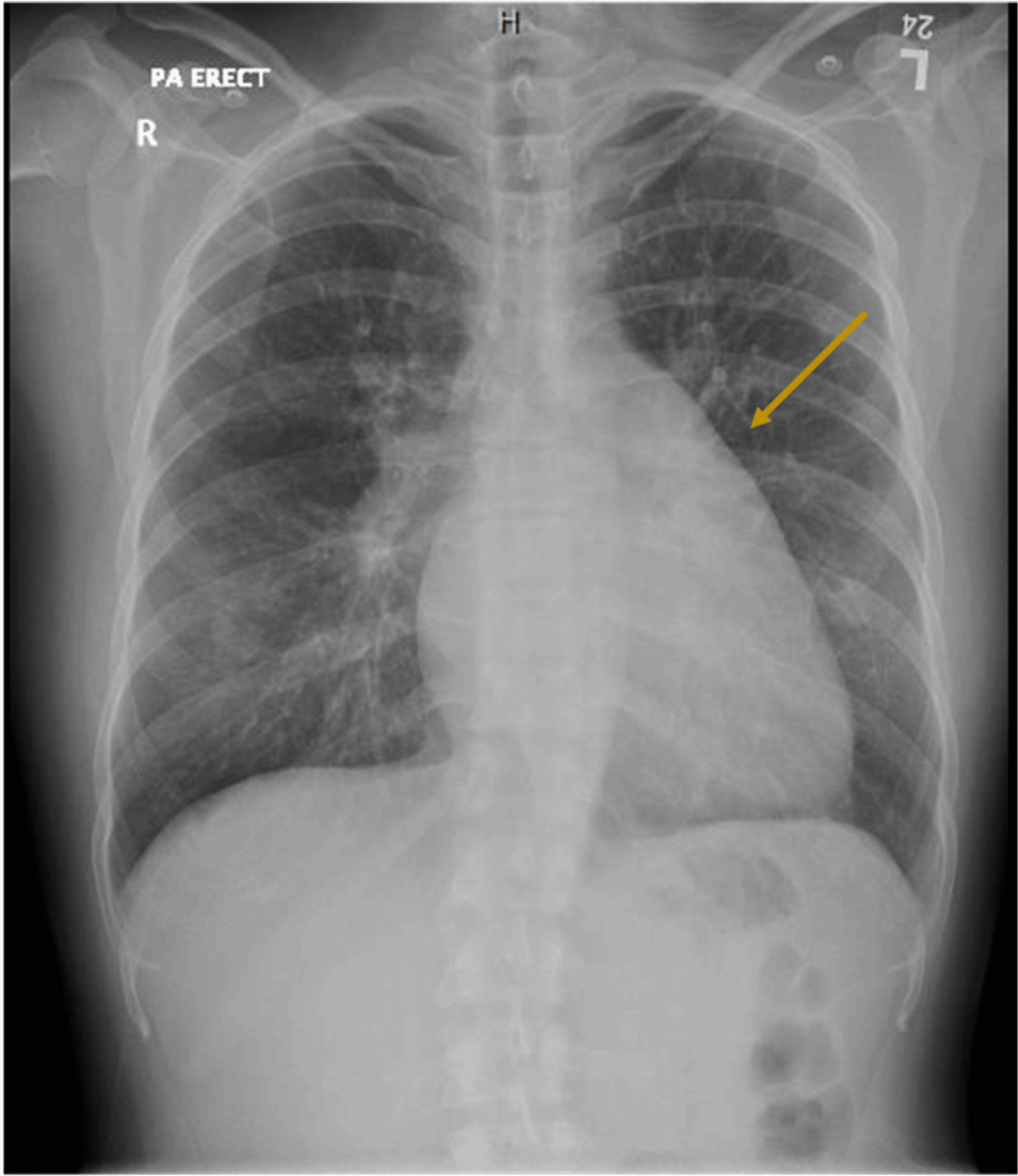
Chest X-ray on her first admission

Initial emergency department laboratory results and chest X-ray (Figure 1) revealed no significant abnormalities apart from an elevated C-reactive protein (CRP) of 141 mg/L. Following initial assessment, the patient was admitted under internal medicine with a working diagnosis of community-acquired pneumonia complicated by hemoptysis. Broad-spectrum antibiotics, piperacillin-tazobactam and azithromycin, were initiated.

During her hospital stay, contrast-enhanced computed tomography (CT) of the thorax on day two revealed diffuse bilateral tree-in-bud nodularity with mediastinal lymphadenopathy, consistent with infective bronchiolitis.

Microbiological investigations revealed positive respiratory viral polymerase chain reaction (PCR) testing for influenza A and respiratory syncytial virus (RSV). Sputum cultures grew *Streptococcus pneumoniae*, *Serratia marcescens*, and *Staphylococcus aureus*, while blood cultures were negative. Given the prolonged constitutional symptoms, pulmonary tuberculosis was considered; however, three sputum acid-fast bacilli (AFB) smears and cultures were negative.

Bronchoscopy performed on day four showed mildly hyperemic airways without endobronchial lesions. Bronchoalveolar lavage (BAL) from the right middle lobe demonstrated predominantly macrophagic cells, while BAL AFB and fungal cultures were negative. Autoimmune screening, including antinuclear antibody (ANA), antineutrophil cytoplasmic antibodies (ANCA), and anti-glomerular basement membrane (anti-GBM) antibodies were also negative.

Antibiotic therapy was de-escalated to oral amoxicillin-clavulanate and oseltamivir for five days. The patient improved clinically with resolution of fever and hemoptysis, while CRP decreased to 33 mg/L. She was discharged on day seven with outpatient follow-up.

Fourteen days after discharge, the patient returned to the emergency department with moderate hemoptysis, estimated at approximately 100 mL over 24 hours, associated with worsening dyspnea occurring even at rest (NYHA Class IV) with associated orthopnea, paroxysmal nocturnal dyspnea, and palpitations. On presentation, she was afebrile with a temperature of 37°C, blood pressure of 118/70 mmHg, heart rate of 118 beats/minute, respiratory rate of 26 breaths/minute, and oxygen saturation of 94% on room air. She appeared acutely dyspneic, and physical examination compared with her previous admission revealed increased bilateral crackles to the mid-lung zones with mild bilateral lower limb pitting edema (1+). On this admission, jugular venous pressure was elevated.

**Figure 2:**
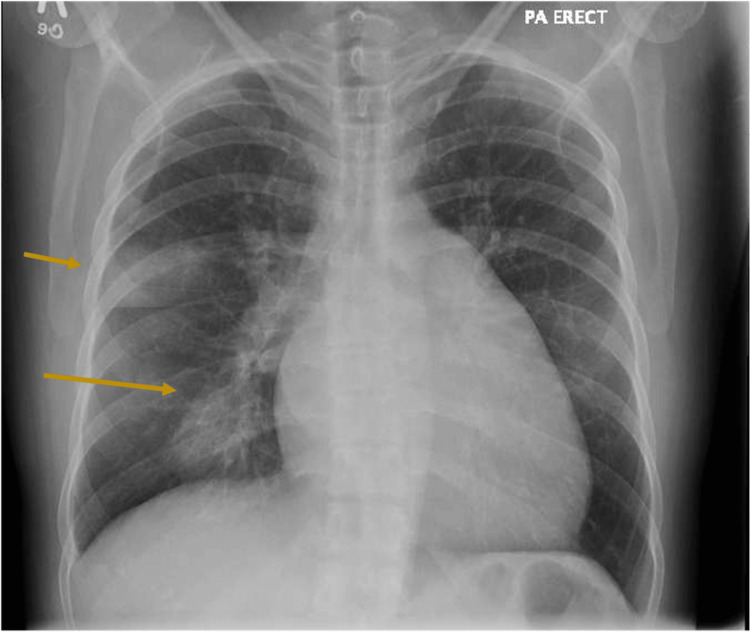
Chest X-ray on the second admission Revealing airspace opacity on the inferior segment of the right upper lobe peripherally and on the right middle lobe.

**Figure 3:**
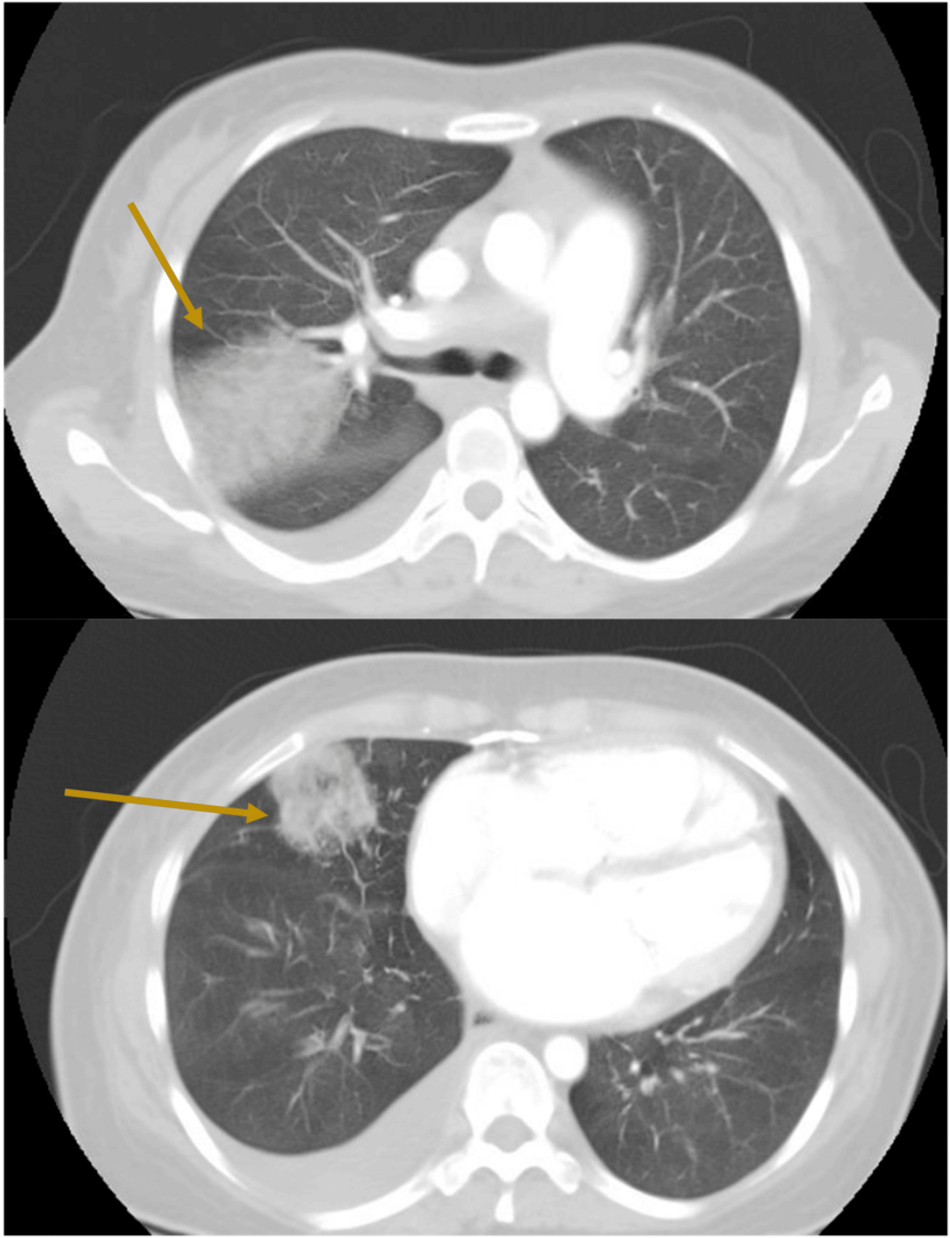
CT chest angiography Showing alveolar haemorrhage and enlarged cardiac size.

A repeat chest radiograph in the emergency department (Figure 2) revealed a new right upper lobe opacity. CT pulmonary angiography the following day (Figure 3), showed multifocal ground-glass alveolar opacities involving the right upper, middle, and lower lobes consistent with alveolar hemorrhage.

**Figure 4:**
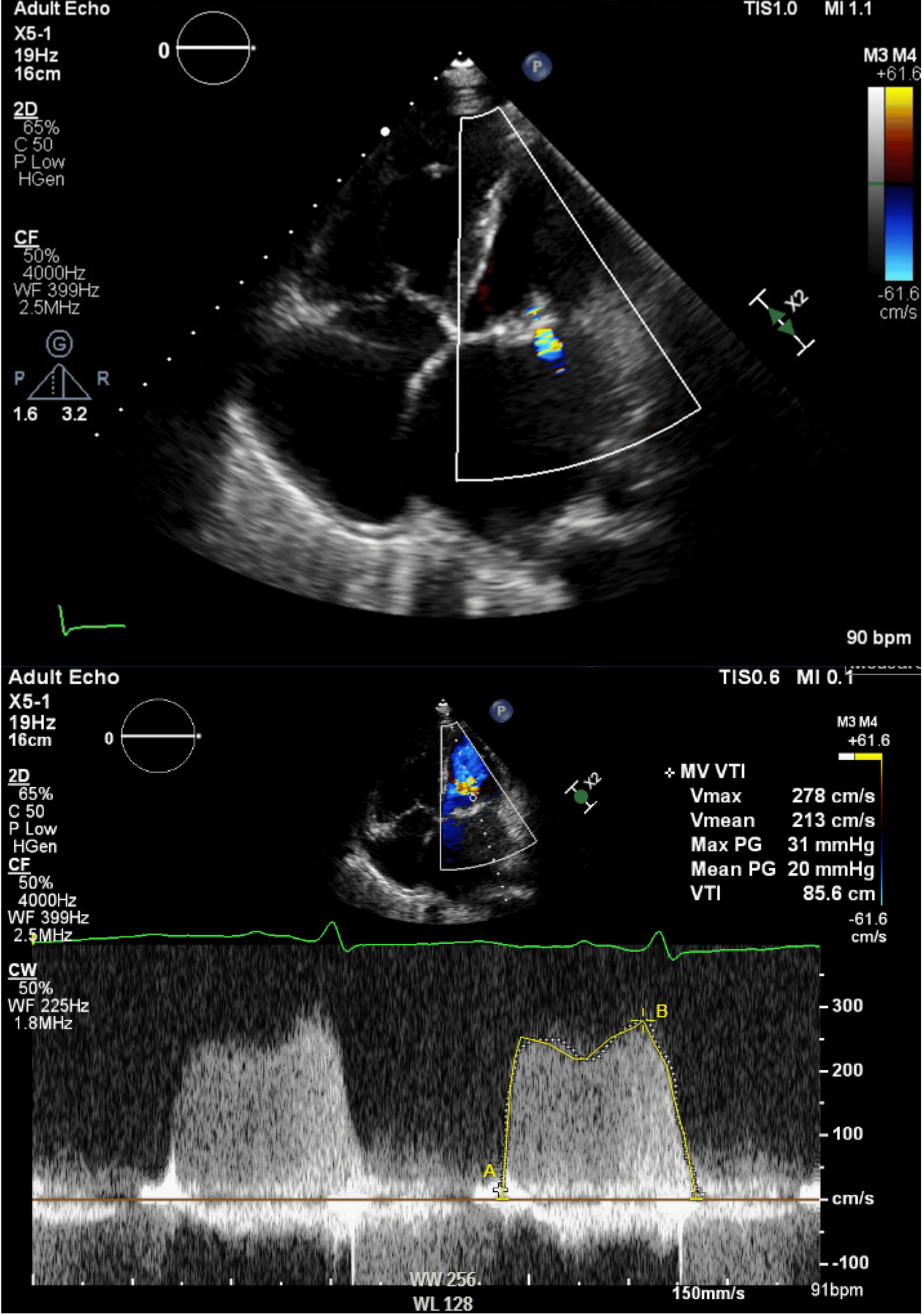
The transthoracic echocardiogram, obtained from the four-chamber apical view Demonstrating severe left atrial dilatation and septal flattening consistent with right ventricular pressure and volume overload. The mitral valve area measured 0.82 cm², with a mean transmitral gradient of 20 mmHg and peak gradient of 31 mmHg, consistent with severe mitral stenosis.

Given recurrent hemoptysis, elevated B-type natriuretic peptide (BNP) of 1,850 pg/mL, and marked left atrial enlargement on imaging, transthoracic echocardiography was performed (Figure 4) and revealed thickened doming mitral valve leaflets with commissural fusion. The mitral valve area (MVA) measured 0.82 cm² by planimetry, and the mean transmitral gradient was 20 mmHg, with a peak gradient of 31 mmHg at a heart rate of 100 beats/minute during the study.

Morphological assessment showed extensive leaflet thickening involving the entire leaflet, calcification extending to the mid-portions of the leaflets, and chordal thickening involving up to one-third of the chordal length, with minimal mobility, all consistent with severe mitral stenosis with a Wilkins score of 13.

The left atrium was severely dilated, while the right atrium was mildly to moderately dilated. The left ventricle was normal in size with preserved systolic function and an estimated ejection fraction of 50-55%. The right ventricle was moderately dilated, and severe tricuspid regurgitation was present, with an estimated pulmonary artery systolic pressure of approximately 98 mmHg, indicating severe pulmonary hypertension.

Upon retrospective review of imaging from the first admission, the initial chest radiograph demonstrated features of mitralization of the cardiac silhouette, a characteristic radiographic finding in mitral stenosis where the left heart border becomes straightened or convex due to left atrial enlargement and prominence of the pulmonary artery. In addition, the initial CT thorax showed marked left atrial enlargement measuring 7 cm. These findings, which were suggestive of underlying mitral stenosis, have been overlooked and not reported.

The final diagnosis was severe rheumatic mitral stenosis causing alveolar hemorrhage due to pulmonary venous hypertension. The patient denied any prior history of rheumatic fever or recurrent pharyngitis. During admission, the patient developed paroxysmal atrial fibrillation on day three, and transesophageal echocardiography (TEE) showed a left atrial appendage thrombus.

Management included intravenous furosemide infusion for decongestion, bisoprolol for rate control, and therapeutic enoxaparin as bridging therapy to warfarin. Cardiology consultation recommended transfer to a tertiary center with cardiovascular surgery capabilities, given the high Wilkins score (13), presence of left atrial thrombus, and severe tricuspid regurgitation, all factors making percutaneous balloon mitral commissurotomy (PMBC) unsuitable.

Subsequently, she underwent bioprosthetic mitral valve replacement using a 31 mm Epic porcine valve with LINX technology, excision of the left atrial appendage with thrombus removal, and tricuspid annuloplasty using a 28 mm annuloplasty ring. The postoperative course was uneventful, and she was discharged in stable condition.

## Discussion

Rheumatic mitral stenosis is typically defined by commissural fusion, leaflet tip restriction, and chordal shortening, with relative pathological sparing of the annulus and the base of the mitral leaflets [4]. It occurs when narrowing of the mitral orifice restricts blood flow from the left atrium to the left ventricle. As the MVA declines below 2.5 cm², left atrial pressure progressively increases, leading to structural remodeling and elevated pulmonary venous pressure. This elevated pulmonary venous pressure causes engorgement and rupture of pulmonary capillaries and bronchial veins, resulting in alveolar hemorrhage and hemoptysis. With ongoing disease progression, pulmonary artery pressures may also increase, resulting in worsening dyspnea and recurrent episodes of hemoptysis [5].

In the present case, given that infections are among the most common causes of hemoptysis [6], the medical team initially pursued an infectious etiology. The polymicrobial sputum cultures (*S. pneumoniae,* *S. marcescens*, and *S. aureus*), alongside positive respiratory viral PCR testing for influenza A and RSV, supported this approach, though some of the positive PCR results may have represented contamination. Nevertheless, a true respiratory infection likely occurred and improved with antibiotic therapy. The antibiotics may have also partially improved pulmonary venous congestion through reduced systemic inflammation rather than treatment of the primary respiratory infection alone, contributing to the transient clinical improvement observed before discharge.

Several diagnostic clues were missed; the patient's six-month history of progressive exertional dyspnea and significant unintentional weight loss should have raised suspicion for chronic cardiopulmonary disease beyond acute infection. More critically, retrospective review revealed radiographic features of mitral stenosis, including mitralization of the heart on chest radiograph and marked left atrial enlargement on CT thorax, that were not initially recognized or reported.

Another reason mitral stenosis was not initially suspected was the absence of an audible murmur. This highlights that, in severe mitral stenosis, the characteristic diastolic murmur may be significantly muffled or even inaudible due to various physiological changes. One contributing factor is the marked enlargement of the right ventricle, which can displace the left ventricle posteriorly, altering the heart's orientation and possibly reducing the transmission of the murmur to the chest wall. Additionally, pulmonary hypertension and right-sided valvular abnormalities can decrease blood flow across the stenotic mitral valve, further diminishing the murmur. Moreover, atrial fibrillation, a common finding in advanced mitral stenosis, abolishes the atrial contraction responsible for the presystolic accentuation of the murmur, thereby reducing its intensity [7]. Extensive calcification of the mitral valve leaflets, often seen in severe cases, can also result in the absence of the characteristic opening snap and diastolic murmur typically associated with mitral stenosis. These factors highlight the need for a comprehensive diagnostic evaluation beyond auscultation when assessing patients with suspected mitral stenosis [8].

Treatment selection in rheumatic mitral stenosis depends on valve morphology, symptom severity, and anatomic suitability for intervention. Percutaneous balloon mitral commissurotomy is the preferred intervention for symptomatic patients with severe mitral stenosis and favorable valve anatomy, defined by low Wilkins scores, absence of significant mitral regurgitation, and absence of left atrial thrombus [9]. This patient's high Wilkins score of 13, presence of left atrial thrombus, and severe tricuspid regurgitation made her unsuitable for percutaneous intervention, necessitating surgical mitral valve replacement with concomitant tricuspid annuloplasty.

The decision to perform bioprosthetic valve replacement rather than mechanical valve replacement likely considered factors including patient preference, anticoagulation risks, and future reproductive considerations, though these details and the indication to perform bioprosthetic instead of mechanical valve replacement were not explicitly stated in the other facility records.

## Conclusions

This case demonstrates how concurrent respiratory infection can mask severe rheumatic mitral stenosis and delay diagnosis despite radiographic evidence of cardiac disease. It also highlights the importance of careful imaging review and considering cardiac causes in young adults presenting with unexplained hemoptysis and dyspnea, particularly when symptoms persist despite appropriate treatment. Early recognition of rheumatic heart disease allows timely intervention and may prevent serious complications such as alveolar hemorrhage and thromboembolism.
